# ﻿*Diaporthe* species (Sordariomycetes, Diaporthales) causing walnut blight and dieback in China

**DOI:** 10.3897/mycokeys.122.158807

**Published:** 2025-09-12

**Authors:** Lili Zhao, Lin Zhang, Yi Ding, Ming Li, Ying Zhang

**Affiliations:** 1 School of Ecology and Nature Conservation, Beijing Forestry University, Beijing, China Beijing Forestry University Beijing China; 2 Intergrated Natural Resources Survey Center, China Geological Survey, Beijing, China Intergrated Natural Resources Survey Center, China Geological Survey Beijing China

**Keywords:** Distribution, novel species, pathogenicity, Sordariomycetes, taxonomy, walnut disease

## Abstract

English walnut (*Juglans regia* L.) is widely cultivated in China due to its economic value and nutritional benefits. Walnut stem blight and dieback is one of the most severe diseases affecting walnut productivity and quality in the country. To clarify the pathogens responsible for walnut stem disease, a comprehensive nationwide survey was conducted. From 276 walnut stem blight and dieback samples collected across seven provinces in China, 292 isolates of *Diaporthe* spp. were obtained. Both morphological characteristics and phylogenetic analyses based on partial ITS, *cal*, *his3*, *tef1-α*, and *tub2* loci were used for fungal identification. Seven species of *Diaporthe* were identified, including one novel species, *D. yunnana*. *Diaporthe* species were most abundant in subtropical southwest China, less common in the temperate north, and absent in Xinjiang. Koch’s postulates confirmed that all seven *Diaporthe* species could cause blight and dieback on walnut branches, with pathogenicity varying significantly among the species. *D. eres* and *D. rostrata* were the most virulent, followed by *D. sackstonii*, *D. amygdali*, *D. citrichinensis*, and *D. yunnana*, while *D. psoraleae-pinnatae* was the least aggressive. This is the first report of *D. citrichinensis*, *D. psoraleae-pinnatae*, and *D. sackstonii* occurring on *J. regia*.

## ﻿Introduction

English walnut (*Juglans regia* L.) is a nut tree species with high nutritional and economic value. Walnut trees are extensively cultivated worldwide, especially in Europe, Asia, and various regions of America (Zhang and Tao 2025). In China, walnut cultivation has a history of more than two thousand years, and China is the largest walnut-producing country in the world, accounting for approximately 48% of global production (Shen et al. 2021; Ting et al. 2023). According to the latest China Forestry and Grassland Statistical Yearbook, China produced nearly 5.93 million tons from over 35.67 million hectares in 2022, with the primary production areas located in Yunnan, Xinjiang, Sichuan, and Shaanxi (Zhang and Tao 2025).

*Diaporthe* (Diaporthaceae, Diaporthales) was typified by *Diaporthe eres* Nitschke (Nitschke 1870). Both generic names, *Diaporthe* and *Phomopsis*, were regularly used. Based on the concept of “one fungus, one name,” *Diaporthe*, being the older generic name, has priority over *Phomopsis* (Rossman et al. 2015). Morphologically, *Diaporthe* is characterized by ostiolate conidiomata, cylindrical phialides, and three types (alpha, beta, and gamma) of conidia (Gomes et al. 2013). Previously, species of *Diaporthe* were identified based on host association, morphology, and cultural characteristics, which led to a large number of species being assigned within the genus (Uecker 1988). Recently, multi-locus phylogenetic analyses have been extensively used for identifying *Diaporthe* species (Guo et al. 2020; Norphanphoun et al. 2022). Norphanphoun et al. (2022) divided the genus into 13 species complexes based on well-supported clades that showed consistent placements in both combined and single-gene trees. However, to facilitate species identification, Dissanayake et al. (2024) reclassified the genus into seven distinct sections. To date, over 1,300 epithets of *Diaporthe* have been listed in Index Fungorum (http://www.indexfungorum.org/; accessed March 2025).

*Diaporthe* species occur as plant pathogens, endophytes, or saprobes on a wide range of hosts, such as *Alnus nepalensis*, citrus, grapevine, sunflower, *Citrus* spp., *Rosa* spp., *Heliconia metallica*, *Heterostemma grandiflorum*, as well as in marine and polluted water environments (Gomes et al. 2013; Yang et al. 2018; Sun et al. 2021; Calabon et al. 2023; Dela Cruz et al. 2025; Li et al. 2025). As plant pathogens, *Diaporthe* spp. can cause a variety of plant diseases, such as root and fruit rots, dieback, stem cankers, leaf spots, blights, seed decay, stem-end rot, shoot blight, branch dieback, and gummosis in various host plants (Gomes et al. 2013; Norphanphoun et al. 2022; Hilário and Gonçalves 2023; López-Moral et al. 2023; Jia et al. 2024; Li et al. 2025). To date, several species of *Diaporthe* have been identified as the main causal agents of walnut disease. For instance, *D. australafricana*, *D. cynaroidis*, *D. eres*, *D. neotheicola*, and *D. rhusicola* have been reported causing dieback of walnut branches and shoots in Chile, southern Spain, California, and the Moravia region of the Czech Republic (Chen et al. 2014; Eichmeier et al. 2020; López-Moral et al. 2020, 2023; Luna et al. 2020). *Diaporthe ampelina*, *D. chamaeropisrhusicola*, *D. eres*, and *D. novem* have been reported to cause walnut canker in the United States (Luna et al. 2023). In China, several species of *Diaporthe* have been reported as causal agents of walnut disease in various regions (Fan et al. 2018; Meng et al. 2018; Wang et al. 2022; Jia et al. 2024). For instance, *D. actinidiicola* has been reported causing branch canker or dieback in many orchards in Henan Province (Cao et al. 2023). *Diaporthe dejiangensis*, *D. juglandigena*, *D. tongrensis*, and *D. hypericin* were isolated from *J. regia* in Guizhou Province (Wang et al. 2022). *Diaporthe amygdali* has been identified as the causal agent of walnut twig canker in Shandong Province (Meng et al. 2018). Three species—*D. eres*, *D. rostrata*, and *D. tibetensis*—have been reported causing canker disease of walnut in Gansu, Beijing, and Tibet (Fan et al. 2015, 2018). Jia et al. (2024) reported that six species, including *D. chaotianensis*, *D. gammata*, *D. olivacea*, *D. tibetensis*, *D. shangluoensis*, and *D. shangrilaensis*, cause walnut branch disease in Shaanxi, Sichuan, and Yunnan provinces.

**Figure 1.**
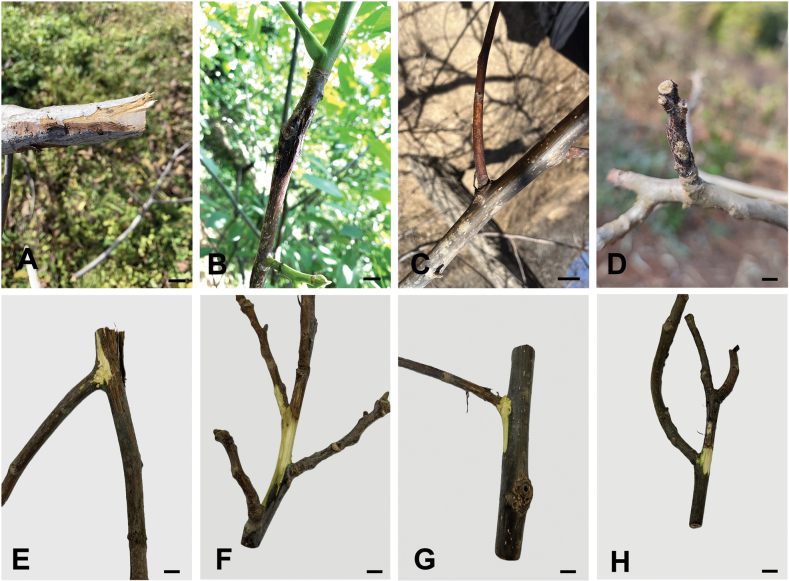
Typical symptoms of walnut blight and dieback on branches caused by *Diaporthe* spp. Scale bars: 1 cm (**A–H**).

Walnut branch disease is a serious problem in China. The infection typically starts on the shoots and then spreads along entire branches. It generally causes discolored areas on the bark, and removing the bark reveals tissues that range from brown to black, eventually leading to the death of the entire branch (López-Moral et al. 2020) (Fig. 1). To clarify the causal agents of walnut branch blight and dieback in China, a screening survey was conducted from 2020 to 2024. The aims of this study were to (i) identify the *Diaporthe* taxa isolated from blighted and dieback branches of walnut, (ii) evaluate the diversity and prevalence of *Diaporthe* associated with walnut in China, and (iii) determine their pathogenicity on walnut.

## ﻿Methods

### ﻿Sample collection and fungal isolation

From 2020 to 2024, a total of 276 samples were collected from diseased or dead branches in walnut plantations across seven sites in China: Beijing, Hebei, Gansu, Shandong, Shanxi, Yunnan, and Xinjiang. Wood fragments (0.5 × 0.5 × 0.2 cm^3^) were aseptically cut from the margin of disease lesions, surface-sterilized with 75% ethanol for 30 seconds, rinsed three times with sterile distilled water, and incubated on Petri dishes containing 2% malt extract agar (MEA) (Zhao et al. 2024). The Petri dishes were incubated in the dark at 28 °C until colonies appeared. Pure cultures were obtained by transferring hyphal tips from the margins of suspected *Diaporthe* colonies onto fresh MEA and incubated in the dark at 28 °C.

### ﻿Morphological characterization

Fungal colonies were initially identified based on morphological characteristics, including colony appearance, conidiomata, conidiogenous cells, and conidia. Colony diameters were measured at 28 °C in darkness on PDA and MEA after 7 days. Colony colors were determined according to Rayner (1970). Additionally, the shapes, colors, and sizes of conidiogenous cells, conidia, and conidiophores were observed under a Nikon Eclipse E600 microscope, and 30–50 conidia were measured to determine their size. Fungal isolates and specimens were deposited at Beijing Forestry University, with duplicates stored at the China General Microbiological Culture Collection Center (CGMCC).

### ﻿DNA extraction, PCR amplification, and sequencing

DNA was extracted from fungal mycelia grown on MEA plates using a CTAB plant genome DNA fast extraction kit (Aidlab Biotechnologies Co., Ltd., Beijing, China). The internal transcribed spacer (ITS) region of ribosomal DNA was amplified and sequenced using primers ITS1 and ITS4 (White et al. 1990); the β-tubulin gene (*tub2*) using primers Bt2a and Bt2b (Glass and Donaldson 1995); the translation elongation factor 1-α gene (*tef1-α*) using primers EF1-728F and EF1-986R (Carbone and Kohn 1999); the calmodulin gene (*cal*) using primers CAL-228F and CAL-737R (Carbone and Kohn 1999); and the histone H3 gene (*his3*) using primers CYLH-3F and H3-1b (Glass and Donaldson 1995). PCR amplification and sequencing followed the protocol of Guo et al. (2020). PCR products were purified and sequenced by BGI Tech Solutions (Beijing Liuhe) Co., Limited (Beijing, China).

### ﻿Phylogenetic analysis

DNA sequences from concatenated ITS, *cal*, *his3*, *tef1-α*, and *tub2* loci were analyzed to investigate the phylogenetic relationships among *Diaporthe* species, using both newly generated sequences and reference sequences retrieved from GenBank (https://www.ncbi.nlm.nih.gov/genbank/) (Suppl. material 1). Sequences were aligned using MAFFT v.7 (Katoh et al. 2019) and edited manually using MEGA v.6.0 (Tamura et al. 2013). Gaps were adjusted manually to optimize the alignment.

Phylogenetic analyses of *Diaporthe* followed the sections proposed by Dissanayake et al. (2024). Maximum likelihood (ML), Bayesian inference (BI), and maximum parsimony (MP) analyses were performed. ML analyses were conducted using RAxML-HPC BlackBox v.8.2.10 (Stamatakis 2014) with the GTR+GAMMA model. MP analysis based on the combined dataset was conducted in PAUP* v.4.0b10 using default settings (Swofford 2002). Ambiguous regions in the alignment were excluded, and gaps were treated as missing data. Clade stability was assessed using bootstrap analysis with 1,000 replicates, with *maxtrees* set to 1,000 and other default parameters (Hillis and Bull 1993). Parsimony scores calculated included consistency index (CI), rescaled consistency index (RC), homoplasy index (HI), and retention index (RI). Bayesian phylogenetic analysis was conducted using MrBayes v.3.2.5 (Ronquist et al. 2012). The best-fit model of nucleotide substitution was selected using the Akaike information criterion (AIC) in MrModeltest v.2.3 (Posada and Buckley 2004). Four Markov Chain Monte Carlo (MCMC) chains were run, with trees sampled every 1,000 generations. Trees were visualized using TreeView v.1.6.6 (Page 1996) and edited in Adobe Illustrator CC2020 (Adobe Systems Inc., USA).

### ﻿Prevalence

To determine the prevalence of *Diaporthe* species obtained in this study, the isolation rate (R^I^) was calculated for each species with the formula R^I^% = (N^s^/N^t^) × 100, where N^s^ was the number of isolates from the same species and N^t^ is the total number of isolates from each sample-collected site (Fu et al. 2019).

The Shannon–Wiener index was used to estimate species diversity at each sampling site, using R version 4.1.2.

### ﻿Pathogenicity testing

The *Diaporthe* isolates obtained in this study were used for pathogenicity testing. Isolates of all species were incubated on MEA plates for 7 days prior to inoculation. The test was performed on lignified, 2-year-old detached walnut branches. The branches were washed, surface-sterilized with 75% ethanol for 1 minute, and the bark surface of each disinfected branch was punctured 20 times with a sterilized inoculating needle within a 10-mm region to a depth of 2 mm (Zhao et al. 2024). An 8-mm-diameter mycelial plug taken from the edge of a fresh colony was placed onto each wounded site. The inoculated area was covered with parafilm. Five replicate branches were inoculated for each isolate, and additional branches were inoculated with fresh MEA agar plugs as controls. All inoculated branches were sealed with parafilm at their ends to prevent desiccation. Pathogenicity was determined by measuring lesion length after three weeks. The data were subjected to analysis of variance (ANOVA), and mean comparisons were conducted using Tukey’s honest significant difference (HSD) test (α = 0.05) in R version 4.1.2.

To fulfill Koch’s postulates, fragments of infected tissue were plated on MEA to re-isolate the fungal isolates, which were identified based on morphological characteristics and DNA sequences.

## ﻿Result

### ﻿Fungal isolation

From 2020 to 2024, 276 samples of diseased or dead walnut branches and trunks were collected from seven sites in China. A total of 292 strains of *Diaporthe* were isolated from these samples, including 103 strains from Beijing, 18 from Gansu, 30 from Hebei, 35 from Shandong, 48 from Shanxi, and 58 from Yunnan, while no strains were obtained from Xinjiang. The occurrence of *Diaporthe* species is shown in Table 2.

### ﻿Phylogenetic analyses

**Figure 2.**
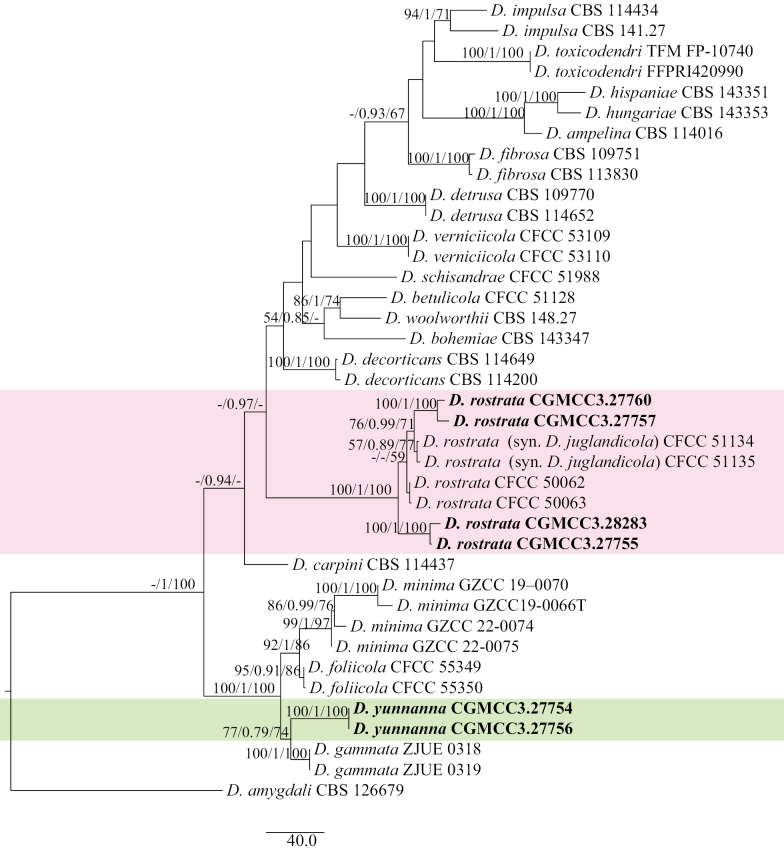
Maximum likelihood (ML) tree generated from sequence analysis of the concatenated ITS, *cal*, *his3*, *tef*1-α, and *tub2* gene dataset of Section Betulicola. RAxML bootstrap support values (ML ≥ 50%), Bayesian posterior probability (PP ≥ 0.70), and maximum parsimony bootstrap support values (MP ≥ 50%) are shown at the nodes (ML/PP/MP).

**Figure 3.**
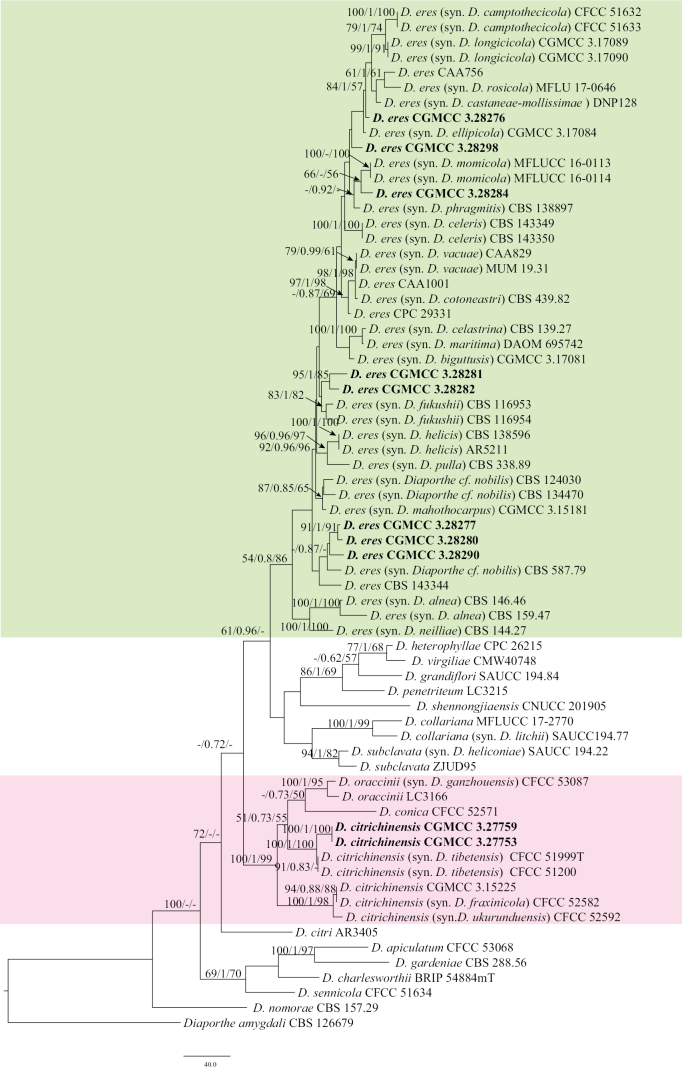
Maximum likelihood (ML) tree generated from sequence analysis of the concatenated ITS, *cal*, *his3*, *tef*1-α, and *tub2* gene dataset of Section Eres. RAxML bootstrap support values (ML ≥ 50%), Bayesian posterior probability (PP ≥ 0.70), and maximum parsimony bootstrap support values (MP ≥ 50%) are shown at the nodes (ML/PP/MP).

**Figure 4.**
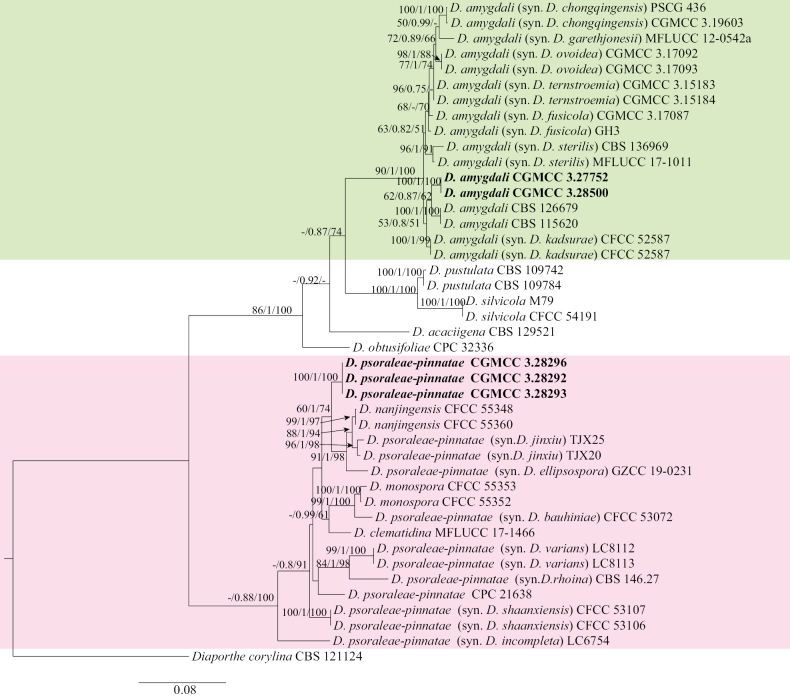
Maximum likelihood (ML) tree generated from sequence analysis of the concatenated ITS, *cal*, *his3*, *tef*1-α, and *tub2* gene dataset of Section Rudis and Psoraleae-pinnatae. RAxML bootstrap support values (ML ≥ 50%), Bayesian posterior probability (PP ≥ 0.70), and maximum parsimony bootstrap support values (MP ≥ 50%) are shown at the nodes (ML/PP/MP).

**Figure 5.**
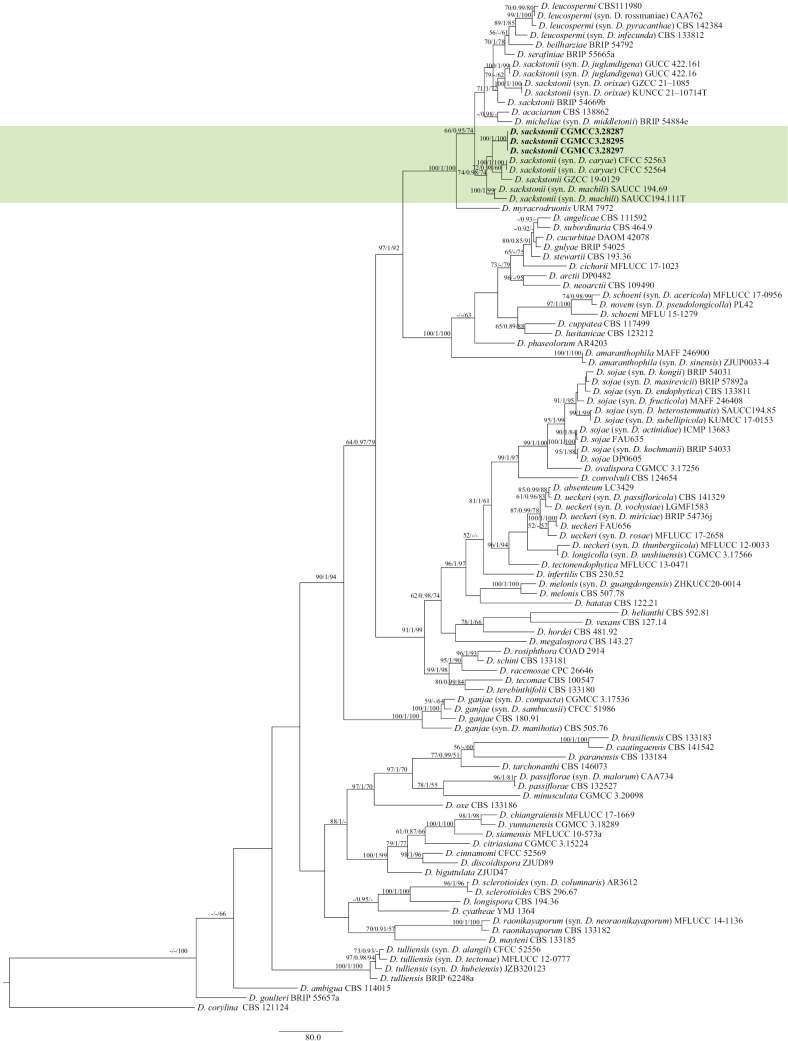
Maximum likelihood (ML) tree generated from sequence analysis of the concatenated ITS, *cal*, *his3*, *tef*1-α, and *tub2* gene dataset of Section Sojae. RAxML bootstrap support values (ML ≥ 50%), Bayesian posterior probability (PP ≥ 0.70), and maximum parsimony bootstrap support values (MP ≥ 50%) are shown at the nodes (ML/PP/MP).

Multi-locus phylogenetic analyses were performed using concatenated ITS, *cal*, *his3*, *tef1-α*, and *tub2* sequences. The *Diaporthe* isolates formed branches representing seven species on the phylogenetic trees, belonging to Section Betulicola, Section Eres, Section Sojae, Section Rudis, and Section Psoraleae-pinnatae (Figs 2–5).

For Section Betulicola, the concatenated ITS, *cal*, *his3*, *tef1-α*, and *tub2* dataset (2,348 characters, with 515 parsimony-informative characters) from 38 ingroup isolates was used for phylogenetic analysis. The outgroup taxon was *D. amygdali* (CBS 126679). The best RAxML tree, with a final likelihood value of –11053.482380, is presented in Fig. 2. RAxML analysis yielded 895 distinct alignment patterns and 11.24% undetermined characters or gaps. For BI analysis, four simultaneous Markov chains were run for 5,100,000 generations. The final average standard deviation of split frequencies was 0.009993. For MP, the heuristic search with random addition of taxa (1,000 replicates) generated 5,000 most parsimonious trees (CI = 0.675, RI = 0.813, RC = 0.549, HI = 0.325). Six isolates clustered in two clades corresponding to *D. rostrata* (four isolates) and *D. yunnana* (two isolates) (Fig. 2).

For Section Eres, the concatenated ITS, *cal*, *his3*, *tef1-α*, and *tub2* dataset (2,262 characters, with 485 parsimony-informative characters) from 67 ingroup isolates was used for phylogenetic analysis. The outgroup taxon was *D. amygdali* (CBS 126679). The best RAxML tree, with a final likelihood value of –13844.510016, is presented in Fig. 3. RAxML analysis yielded 973 distinct alignment patterns and 16.18% undetermined characters or gaps. For BI analysis, four simultaneous Markov chains were run for 2,000,000 generations. The final average standard deviation of split frequencies was 0.009935. The heuristic search with random addition of taxa (1,000 replicates) generated 5,000 most parsimonious trees (CI = 0.556, RI = 0.706, RC = 0.393, HI = 0.444). Ten isolates clustered in two clades corresponding to *D. eres* (eight isolates) and *D. citrichinensis* (two isolates) (Fig. 3).

For Sections Rudis and Psoraleae-pinnatae, the concatenated ITS, *cal*, *his3*, *tef1-α*, and *tub2* dataset (2,249 characters, with 628 parsimony-informative characters) from 42 ingroup isolates was used for phylogenetic analysis. The outgroup taxon was *D. corylina* (CBS 121124). The best RAxML tree, with a final likelihood value of –12244.597254, is presented in Fig. 4. RAxML analysis yielded 1,009 distinct alignment patterns and 17.85% undetermined characters or gaps. For BI analysis, four simultaneous Markov chains were run for 1,500,000 generations. The final average standard deviation of split frequencies was 0.009051. The heuristic search with random addition of taxa (1,000 replicates) generated 5,000 most parsimonious trees (CI = 0.718, RI = 0.911, RC = 0.654, HI = 0.282). Five isolates clustered in two clades corresponding to *D. amygdali* (two isolates) and *D. psoraleae-pinnatae* (three isolates) (Fig. 4).

For Section Sojae, the concatenated ITS, *cal*, *his3*, *tef1-α*, and *tub2* dataset (2,408 characters, with 984 parsimony-informative characters) from 104 ingroup isolates was used for phylogenetic analysis. The outgroup taxon was *D. corylina* (CBS 121124). The best RAxML tree, with a final likelihood value of –32296.521228, is presented in Fig. 5. RAxML analysis yielded 1,484 distinct alignment patterns and 25.65% undetermined characters or gaps. For BI analysis, four simultaneous Markov chains were run for 7,500,000 generations. The final average standard deviation of split frequencies was 0.009634. The heuristic search with random addition of taxa (1,000 replicates) generated 5,000 most parsimonious trees (CI = 0.374, RI = 0.693, RC = 0.259, HI = 0.626). Three isolates clustered in one clade corresponding to *D. sackstonii* (three isolates) (Fig. 5).

### ﻿Taxonomy

#### ﻿ Diaporthe amygdali

Taxon classificationFungiDiaporthalesDiaporthaceae

(Delacr.) Udayanga, Crous, K.D. Hyde, Fungal Divers 56: 166 (2012)

21639321-B710-5457-BDFA-1F898FAFEA2E

Suppl. material 2

##### Specimens examined.

China • Yunnan Province, Kunming City, from diseased branches of *J. regia*., Y. Ding, M. Li and L.L. Zhao, 23 February 2024 (YN-6, culture CGMCC3.27752; YN-2, culture CGMCC3.28500).

##### Notes.

*Diaporthe amygdali* was first described as *Fusicoccum amygdali* Delacr., causing cankers on almonds in France (Delacroix 1905). Udayanga et al. (2012) assigned *F. amygdali* to *Diaporthe* as *D. amygdali*. Dissanayake et al. (2024) verify *D. amygdali* is a single species rather than a species complex. Phylogenetically, two isolates clustered together with the ex-type isolates of *D. amygdali* CBS 126679 with middle statistical (62%/0.87/62%) (Fig. 4); the base pair similarity shows 98.9% (464/469) on *cal*, 99.3% (449/452) on *his*, 99% (519/524) on ITS, 99.4% (327/329) on *tef1-α*, and 99.8% (469/470) on *tub2* compared to the ex-type of *D. amygdali*. Based on sequence data and morphology, these isolates were confirmed to belong to *D. amygdali* (Table 1). Thus far, *D. amygdali* has been reported to cause walnut branch disease in Southern Spain and Shandong province in China (Meng et al. 2018, López-Moral et al. 2020). In this study, *D. amygdali* were isolated from Yunnan Province.

**Table 1.** Morphological characteristics of known *Diaporthe* species.

Species	Conidiomata	Conidiophores	Conidiogenous cells	Alpha conidia	Beta conidia	Gamma conidia
*D. amygdali*	Pycnidial globose or irregular, solitary or aggregated, wrapped in hyphae embedded colony surface, white to brown.	hyaline, subcylindrical, densely aggregated, 7.0–18.5 × 1.5–4.0 μm.	phialidic, hyaline, cylindrical, straight or slightly curved, 1.5–2.5 μm, tapered towards the apex.	Not observed.	hyaline, aseptate, filiform, curved, tapering towards end, 23.5–35.0 × 1.0–2.0 μm.	Not observed.
*D. citrichinensis*	Pycnidial irregular, solitary or aggregated, brown to dark brown	cylindrical, hyaline, densely aggregated, 6.0–10.5 × 1.0–3.0 μm.	phialidic, cylindrical, 5.0–11.0 × 1.5–2.5 μm, tapered towards the apex	aseptate, fusoid with obtuse ends, hyaline, biguttulate 6.0–10.0 × 1.5–3.0 μm.	filiform, hyaline, aseptate, slightly curved at one end and both ends rounded, 32.0–44.0 × 1.0–2.0 μm.	Not observed.
*D. eres*	Pycnidial solitary or aggregated, with yellowish or white translucent conidial drops exuded from the ostioles.	hyaline, smooth, unbranched, ampulliform, straight to sinuous, 9.0–16 × 2.0–3.0 μm.	phialidic, cylindrical, terminal, slightly, 5.0–1.5 μm diam tapering towards the apex	aseptate, hyaline, smooth, ovate to ellipsoidal, biguttulate, 6.0–9.0 × 2.5–3.0 μm.	aseptate, hyaline, smooth, fusiform to hooked, 23.0–32.0 × 1.0–2.0 μm.	Not observed.
*D. psoraleae-pinnatae*	Pycnidial irregular shape, solitary or aggregated, white or yellowish translucent conidial drops exuded from the ostioles.	hyaline, smooth, densely aggregated, ampulliform, 9.5–23.0 × 2–6 μm.	phialidic, hyaline, terminal, cylindrical, straight or slightly curved, 6.0–19.0 × 1.5–2.5 μm, tapered towards the apex.	aseptate, fusiform, biguttulate, apex subobtuse, 7.5–10.5 × 2.0–3.0 μm.	hyaline, aseptate, guttulate, filiform, curved, 32.0–48.0 × 1.0–2.5 μm.	Not observed.
*D. rostrata*	Pycnidial solitary or aggregated, wrapped in hyphae embedded on colony surface, with yellowish translucent conidial drops exuded from the ostioles.	hyaline, smooth, densely aggregated, 12.0–21.0 × 2–4.5 μm.	Conidiogenous cells phialidic, hyaline, terminal, cylindrical, straight, 5.5–8.5 × 2.0–3.5 μm.	hyaline, smooth, aseptate, fusiform to oval, biguttulate or multi-guttulate, 7.0–10.0 × 4.0–5.0 μm.	Not observed.	hyaline, guttulate, smooth, aseptate, with only one acute end, 10.5–13.0 × 2.5–4.0 µm.
*D. sackstonii*	Pycnidial globose, solitary or aggregated, wrapped in hyphae embedded on MEA colony surface, white to brown.	hyaline, smooth, unbranched, densely aggregated, ampulliform, 12.5–37 × 2.0–4.0 μm.	Conidiogenous cells phialidic, hyaline, terminal, cylindrical, straight, 7.5–17 × 1.5–2.5 μm, tapered towards the apex.	hyaline, aseptate, fusiform to oval, obtuse at both ends, 5.5–7.5 × 2.5–3.5 μm.	hyaline, aseptate, multi-guttulate, filiform, curved, tapering towards both ends, 27.0–39.0 × 1.0–2.5 μm.	Not observed.

**Table 2.** Number of isolates collected for each *Diaporthe* species identified and sites investigated in this study.

	Beijing	Gansu	Hebei	Shandong	Shanxi	Yunnan	Xinjiang	Total
*D. amygdali*	-	-	-	-	-	5	-	5 (1.7%)
*D. citrichinensis*	-	-	-	-	-	15	-	15 (5.1%)
*D. eres*	81	17	10	31	32	26	-	197 (67.5%)
*D. psoraleae-pinnatae*	8	-	-	-	-	-	-	8 (2.7%)
*D. rostrata*	7	1	20	2	16	6	-	52 (17.8%)
*D. sackstonii*	7	-	-	-	-	-	-	7 (2.4%)
*D. yunnana*	-	-	-	2	-	6	-	8 (2.7%)
Total	103	18	30	35	48	58	0	
Shannon-Wiener index	0.75	0.21	0.64	0.43	0.64	1.40	0	

#### ﻿ Diaporthe citrichinensis

Taxon classificationFungiDiaporthalesDiaporthaceae

F. Huang, K.D. Hyde & H.Y. Li, Fungal Divers 61: 247 (2013)

C020B2EA-A5A6-5ABA-8ACF-3BF0E22494C1

Suppl. material 3

##### Specimens examined.

China • Yunnan Province, Kunming City, from diseased branches of *J. regia*, Y. Ding, M. Li and L.L. Zhao, 23 February 2024 (YN-7, culture CGMCC3.27759; YN-26, culture CGMCC3.27753).

##### Notes.

*Diaporthe citrichinensis* was first described from decaying wood of *Citrus unshiu* in China (Huang et al. 2013). Dissanayake et al. (2024) treated *Diaporthe acerigena*, *D. albosinensis*, *D. coryli*, *D. fraxinicola*, *D. tibetensis*, and *D. ukurunduensis* as the synonyms of *D. citrichinensis*. Phylogenetically, two isolates clustered together with *D. citrichinensis* with high support (100%/1/100%) (Fig. 3); the base pair similarity shows 100% (471/471) on *cal*, 97.7% (434/444) on *his*, 100% (530/530) on ITS, 100% (333/333) on *tef1-α*, and 100% (480/480) on *tub2* compared to the ex-type of *D. citrichinensis*. Morphologically, alpha and beta conidia are similar to *D. citrichinensis* (6.0–10.0 × 1.5–3.0 vs. 5.5–9 × 1.5–2.5 μm) and (32–44 × 1–2 vs. 27.5–40 × 1–1.5 μm) (Table 1) (Huang et al. 2013). In this study, *D. citrichinensis* was collected from the walnut plantation of Yunnan. This is the first report of *D. citrichinensis* occurring on *J. regia*.

#### ﻿ Diaporthe eres

Taxon classificationFungiDiaporthalesDiaporthaceae

Nitschke, Pyrenomyc. Germ. 2: 245 (1870)

6377FFB3-4738-5675-AAAD-75C22128690B

Suppl. material 4

##### Specimens examined.

China • Beijing City, from diseased branches of *J. regia*., Y. Zhang, L.L. Zhao and L. Zhang, 14 December 2021 (2021-JF-6, culture CGMCC3.28277; CGMCC 3.28281; 2021-JF-10, culture CGMCC3.28282, CGMCC3.28284). • Shanxi Province, Jiaokou City, from diseased branches of *J. regia*, Y. Ding, M. Li and L.L. Zhao, 28 February (JK-4, culture CGMCC3.28276). • Yunnan Province, Kunming City, from diseased branches of *J. regia*, Y. Ding, M. Li and L.L. Zhao, 23 February 2024 (YN-23, culture CGMCC3.28290). • Shandong Province, Liaocheng City, from diseased branches of *J. regia*, Y. Ding, M. Li and L.L. Zhao, 5 February 2024 (LC-12, culture CGMCC 3.28280). • Hebei Province, Chengde City, from diseased branches of *J. regia*, Y. Ding, M. Li and L.L. Zhao, 13 February 2024 (CD-3, culture CGMCC3.28298).

##### Notes.

*Diaporthe eres* was first described by Nitschke (1870) and collected from *Ulmus* sp. in Germany. It has a wide distribution and a broad host range as a pathogen, endophyte, or saprobe, and can cause a variety of plant diseases (Udayanga et al. 2014). Hilário et al. (2021b) and Dissanayake et al. (2024) identified the *D. eres* complex as a single species, *D. eres*. Phylogenetically, eight isolates clustered within *D. eres* (Fig. 3). Therefore, these isolates were confirmed to belong to *D. eres*, based on sequence data and morphology (Table 1). In this study, more than half of the isolates (189, 67.5%) belong to *D. eres*, which is nationally distributed in Beijing, Gansu, Hebei, Shandong, Shanxi, and Yunnan, causing walnut branch diseases.

#### ﻿ Diaporthe psoraleae-pinnatae

Taxon classificationFungiDiaporthalesDiaporthaceae

Crous & M.J. Wingf., Persoonia 31: 205 (2013)

E8B9C59B-12A7-5709-9095-02D5D4F7FCDC

Suppl. material 5

##### Specimens examined.

China • Beijing, Changping District, Heishanzhai Village, from branches of *J. regia*, Y. Zhang, L.L. Zhao and L. Zhang, 26 August 2022 (HSZ-1, culture CGMCC3.28292; HSZ-5, culture CGMCC3.28293, CGMCC3.28296).

##### Notes.

*Diaporthe psoraleae-pinnatae* was first described from dieback branches of *Psoralea pinnata* in South Africa (Crous et al. 2013). Recently, the entire section Psoraleae-pinnatae has been identified as a single species and named *Diaporthe psoraleae-pinnatae*, with *D. aquatica*, *D. bauhiniae*, *D. ellipsospora*, *D. incomplete*, *D. jinxiu*, *D. rhoina*, *D. shaanxiensis*, and *D. varians* all treated as its synonyms (Dissanayake et al. 2024). Phylogenetically, three isolates clustered together with *D. psoraleae-pinnatae* (Fig. 4); the base pair similarity shows 100% (469/469) on *cal*, 97.7% (452/452) on *his*, 97% (508/524) on ITS, 100% (329/329) on *tef1-α*, and 100% (470/470) on *tub2* compared to the ex-type of *D. psoraleae-pinnatae*. Morphologically, conidiogenous cells and alpha conidia are similar to the ex-type isolate of *D. psoraleae-pinnatae* (6–19 × 1.5–2.5 vs. 8–15 × 2–3 μm) and (7.5–10.5 × 2.0–3.0 vs. 9–10 × 2.5–3 μm) (Table 1) (Crous et al. 2013). In this study, *D. psoraleae-pinnatae* was collected from Beijing. This is the first report of *D. psoraleae-pinnatae* occurring on *J. regia*.

#### ﻿ Diaporthe rostrata

Taxon classificationFungiDiaporthalesDiaporthaceae

C.M.Tian, X.L.Fan & K.D.Hyde, Mycol. Progr. 14: 82 (2015)

17E70BFC-37A4-5B25-82F8-0C916B3665E3

Suppl. material 6

##### Material examined.

China • Beijing City, Haidian District, from diseased branches of *J. regia*, M. Li, L.L. Zhao and L. Zhang, 19 October 2020 (JF-11, ex-type culture CGMCC 3.28283); Hebei Province, Chengde City, from diseased branches of *J. regia*, M. Li and L.L. Zhao, 13 February 2024 (CD-22, culture CGMCC3.27755); Shanxi Province, Jiaokou City, from diseased branches of *J. regia*, Y. Ding, M. Li and L.L. Zhao, 28 February 2024 (JK-14-2, culture CGMCC3.27757; JK-16-2, culture CGMCC3.27760).

##### Notes.

*Diaporthe rostrata* was first described from *Juglans mandshurica* in Gansu Province, China (Fan et al. 2015). Dissanayake et al. (2024) compared morphological details and phylogenetic analysis, treating *D. juglandicola* as the synonym of *D. rostrata*. Phylogenetically, four isolates clustered together with *D. rostrata* with high support (100%/1/100%) (Fig. 2); the base pair similarity shows 99.6% (455/457) on *cal*, 98.2% (439/447) on *his*, 100% (550/550) on ITS, 99.2% (357/360) on *tef1-α*, and 100% (473/473) on *tub2* compared to the ex-type of *D. rostrata*. Morphologically, the culture characteristics and alpha conidia are consistent with the description of *D. rostrata* (Table 1) (Fan et al. 2015). In this study, *D. rostrata* was collected from the walnut plantations of Beijing, Gansu, Hebei, Shandong, Shanxi, and Yunnan provinces.

#### ﻿ Diaporthe sackstonii

Taxon classificationFungiDiaporthalesDiaporthaceae

R.G. Shivas, S.M. Thomps. & Y.P. Tan, Persoonia 35: 46 (2015)

7486EB78-95E2-56F3-8E5D-47E2A4BAF678

Suppl. material 7

##### Material examined.

China • Beijing City, Haidian District, JiuFeng forest farm, from branches of *J. regia*, M. Li, L.L. Zhao and L. Zhang, 26 August 2022 (2022-JF-34, culture CGMCC3.28287, CGMCC3.28295, CGMCC3.28297).

##### Notes.

*Diaporthe sackstonii* was first described from *Helianthus annuus* in Australia (Thompson et al. 2015). Pereira and Phillips (2024) treated *D. caryae*, *D. machili*, *D. juglandigena*, and *D. orixae* as the synonyms of *D. sackstonii*. Phylogenetically, three isolates clustered together with *D. sackstonii* (Fig. 5); the base pair similarity shows 97.5% (466/478) on *his*, 99.2% (506/510) on ITS, 98.1% (370/377) on *tef1-α*, and 98.6% (488/495) on *tub2* compared to the ex-type of *D. sackstonii*. Morphologically, the alpha conidia are similar to *D. sackstonii* (5–7.5 × 2.5–3.5 vs. 6–7 × 2–2.5) (Table 1) (Thompson et al. 2015). In this study, *D. sackstonii* was collected from the walnut plantation of Beijing. This collection is the first report of *D. sackstonii* occurring on *J. regia*.

#### ﻿ Diaporthe yunnana

Taxon classificationFungiDiaporthalesDiaporthaceae

Y. Zhang ter & L.L. Zhao sp. nov.

8CA9603C-35C6-59B1-BBEC-2F7751FE5BA6

MycoBank No: 854968

Fig. 6

##### Etymology.

Named after the place, Yunnan, where the fungus was abundantly found.

##### Description.

***Sexual morph***: not observed. ***Asexual morph*: *Conidiomata*** pycnidial, produced on PDA, globose or irregular, solitary, dark brown to black, 290–810 μm diam. ***Conidiophores*** hyaline, smooth, densely aggregated, 12–20.5 × 1.5–3 μm; ***Conidiogenous cells*** phialidic, hyaline, terminal, cylindrical, 5.5–10 × 1.5–2.5 μm diam, tapered towards the apex. ***Alpha conidia*** hyaline, aseptate, ellipsoid to cylindrical, obtuse at both ends, multi-guttulate, 6–10.5 × 2–3 μm (mean ± SD = 8.5 ± 1.0 × 2.8 ± 0.2 μm, n = 30). ***Beta conidia*** hyaline, aseptate, filiform, curved, tapering towards both ends, multi-guttulate, 25.5–42 × 1–1.7 μm (mean ± SD = 34.5 ± 3.6 × 1.4 ± 0.2 μm, n = 30). ***Gamma conidia*** infrequent, hyaline, aseptate, botuliform, tapering towards both ends, multi-guttulate, 12.5–18 × 2–2.5 μm (mean ± SD = 14.0 ± 1.5 × 1.9 ± 0.1 μm, n = 30).

**Figure 6.**
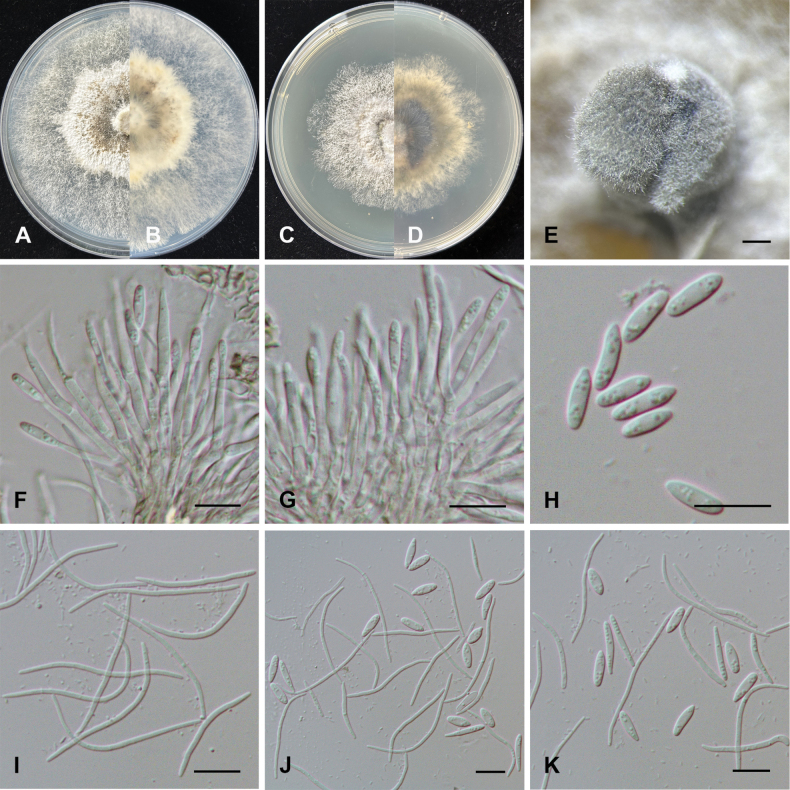
Morphological characteristics of *Diaporthe yunnana*. **A, B.** Colonies and reverse after 7 days on PDA; **C, D.** Colonies and reverse after 7 days on MEA; **E.** Conidiomata; **F, G.** Conidiophores and conidiogenous cells; **H.** Alpha conidia; **I.** Beta conidia; **J, K.** Alpha, beta, and gamma conidia. Scale bars: 500 μm (**E, F**); 10 μm (**G–K**).

##### Culture characteristics.

On PDA, colony at first flat with white felty mycelium, becoming brown in the center, flourishing at center of colony, reverse white to brown. On MEA, white on surface, reverse white to dark brown. Colonies cover the Petri dish diameter on PDA and reach 57 mm in diameter on MEA.

##### Material examined.

China • Yunnan Province, Kunming City, from diseased branches of *J. regia*, Y. Ding, M. Li and L.L. Zhao, 23 February 2024 (holotype YN-12, ex-type culture CGMCC3.27754; other culture CGMCC3.27756).

##### Notes.

Multi-locus phylogenetic analysis indicated that *Diaporthe yunnana* formed a moderately supported subclade with *D. gammata* (77%/0.79/74%) (Fig. 2). Morphologically, *D. yunnana* can be readily distinguishable from *D. gammata* by its shorter beta conidia (25.5–42 × 1–1.7 vs. 29–48.5 × 1–2 μm) and smaller-size gamma conidia (12.4–18.2 × 1.8–2.3 vs. 16–31.5 × 1.5–4 μm) (Xiao et al. 2023). Based on nucleotide base comparison, *D. yunnana* can be distinguished from *D. gammata* by base differences as follows: 13/548 bp for ITS (2.37%), 18/440 bp for *his3* (4.08%), 23/360 bp for *tef1-α* (6.39%), and 8/486 bp for *tub2* (1.65%) (Xiao et al. 2023).

### ﻿Prevalence

**Figure 7.**
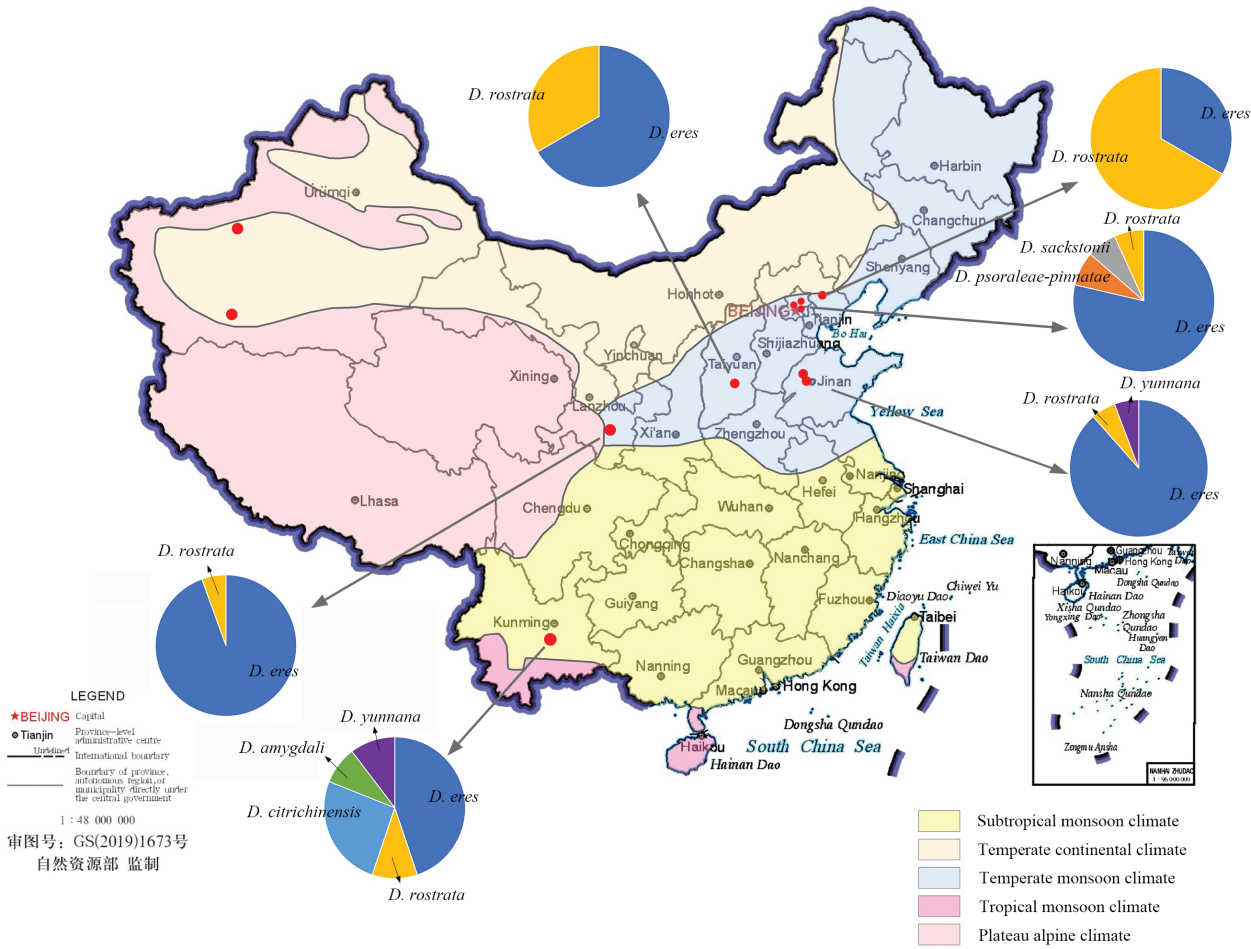
Map of China indicating locations where walnut trees were sampled and the species of *Diaporthe* obtained from each site. The seven species of *Diaporthe* are indicated.

Prevalence analysis revealed that *Diaporthe eres* was the dominant species (67.5%), followed by *D. rostrata*, *D. citrichinensis*, *D. psoraleae-pinnatae*, *D. yunnana*, *D. sackstonii*, and *D. amygdali*. Among them, *D. eres* was the most prevalent species in Beijing, Gansu, Shandong, Shanxi, and Yunnan, while *D. rostrata* was dominant in Hebei (Table 2, Fig. 7).

Further analysis of *Diaporthe* species prevalence across the sampling areas showed that fewer species were identified in the northern regions with a temperate monsoon climate, whereas greater diversity was observed in the southwestern regions with a subtropical monsoon climate. In the northwestern regions with a temperate continental climate, *Diaporthe* species were not isolated (Table 2, Fig. 7).

### ﻿Pathogenicity testing

**Figure 8.**
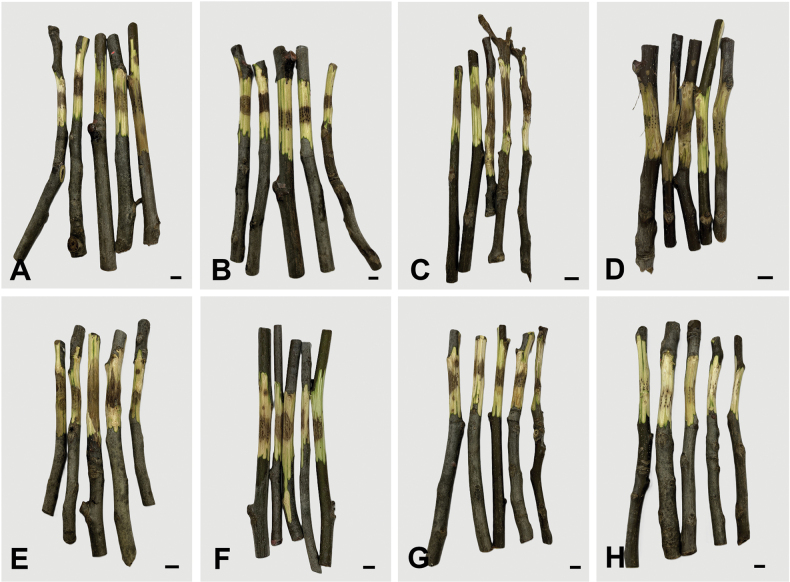
Symptoms of seven *Diaporthe* species inoculated on walnut branches after three weeks. **A.** Inoculated *D. amygdali*; **B.** Inoculated *D. citrichinensis*; **C.** Inoculated *D. eres*; **D.** Inoculated *D. psoraleae-pinnatae*; **E.** Inoculated *D. rostrata*; **F.** Inoculated *D. sackstonii*; **G.** Inoculated *D. yunnana*; **H.** CK. Scale bars: 1 cm (**A–H**).

**Figure 9.**
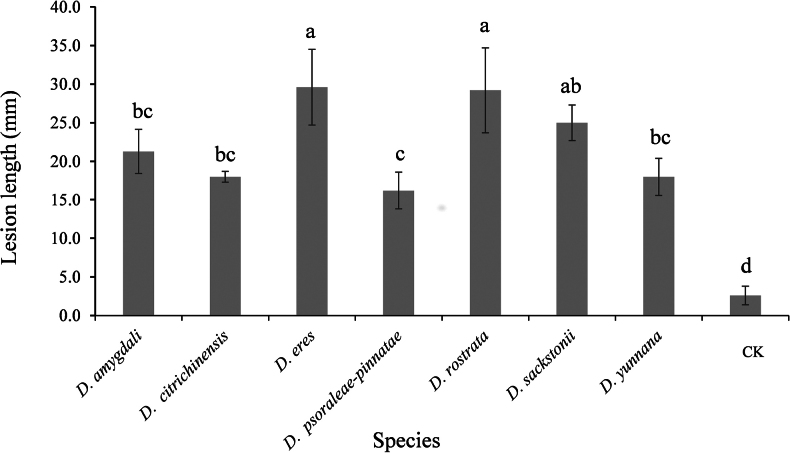
Lesion length caused by *Diaporthe* species inoculated on walnut detached stems. Columns represent the mean values of five replicate branches. A vertical bar with different letters indicates significantly different (*p* < 0.05).

All *Diaporthe* species tested in this study were pathogenic on walnut branches. Brown lesions appeared at the inoculation sites three weeks after inoculation, while no symptoms were observed in the control treatment (Fig. 8). The average lengths of the necrotic lesions caused by *D. eres* (29.6 ± 4.9 mm) and *D. rostrata* (29.2 ± 5.5 mm) were significantly greater than those caused by *D. amygdali* (21.3 ± 2.9 mm), *D. citrichinensis* (18.0 ± 0.7 mm), *D. psoraleae-pinnatae* (16.2 ± 2.4 mm), and *D. yunnana* (18.0 ± 2.4 mm). There was no significant difference among *D. eres*, *D. rostrata*, and *D. sackstonii*. The lesion length caused by *D. sackstonii* (25.0 ± 2.3 mm) was significantly greater than that caused by *D. psoraleae-pinnatae*. No significant differences were observed among *D. amygdali*, *D. citrichinensis*, *D. psoraleae-pinnatae*, and *D. yunnana* (Fig. 9).

## ﻿Discussion

Among the *Diaporthe* species isolated from walnut, *D. eres* was the most prevalent in this study, comprising 67.5% of all *Diaporthe* isolates. Similar results were reported by Fan et al. (2018), who collected infected walnut branches in Beijing, Gansu, Henan, Ningxia, Sichuan, and Tibet, showing that most strains were *D. eres*, occurring in five provinces. Guo et al. (2020) also reported that *D. eres* was the most prevalent species causing pear shoot canker in China, accounting for 54.7% of all *Diaporthe* isolates. Moreover, *D. eres* was identified as the most prominent species associated with grapevine dieback in China, representing 37.5% of total isolates (Manawasinghe et al. 2019). *Diaporthe citri*, however, was the dominant species associated with *Citrus* in southern China (Xiao et al. 2023), possibly indicating that *Diaporthe* species composition varies across different host plants.

Climate types largely influenced the species diversity of *Diaporthe*. Yunnan Province, characterized by a subtropical monsoon climate, exhibited the highest species diversity, followed by Beijing, Shanxi, Hebei, Shandong, and Gansu, which are mainly temperate monsoon regions. No *Diaporthe* species were found in Xinjiang, which has a temperate continental climate. Jia et al. (2024) isolated six species from twigs of *Juglans regia* in Shaanxi, Sichuan, and Yunnan. Of these, five species were collected from Sichuan and Yunnan, which are both characterized by a subtropical monsoon climate. Similar findings were reported by Guo et al. (2020), who observed higher species diversity in the southern Yangtze River region (subtropical monsoon climate) compared to the northern region (temperate monsoon climate). However, no *Diaporthe* species were found in Gansu, Shanxi, and Xinjiang, likely due to the predominantly temperate continental climate in these areas (Guo et al. 2020). Xiao et al. (2023) collected citrus disease samples in southern China and reported significant *Diaporthe* diversity, particularly in Hunan Province. This may be because the southern climate is humid and warm—conditions suitable for the survival and prevalence of *Diaporthe* species—whereas drought and extremely low temperatures prevail in northern and northwestern China, especially in Xinjiang, making these regions unsuitable for *Diaporthe*.

Pathogenicity tests showed that all seven species retrieved in this study were causal agents of walnut branch blight and dieback, producing dark brown necrosis at the inoculation sites. However, pathogenicity varied significantly among species. *Diaporthe eres*, *D. rostrata*, and *D. sackstonii* exhibited the greatest severity on walnut branches, followed by *D. amygdali*, *D. citrichinensis*, and *D. yunnana*, while *D. psoraleae-pinnatae* was the least aggressive. A similar result was obtained by Jia et al. (2024), who reported that six species were associated with walnut branch and twig cankers. Their pathogenicity tests revealed that all six species could cause disease on walnut, with *D. shangrilaensis*, *D. olivacea*, and *D. shangluoensis* being more aggressive, followed by *D. chaotianensis* and *D. tibetensis*, while *D. gammata* was the least aggressive. Previous studies have demonstrated that the pathogenicity of *Diaporthe* species varies on a single host (Manawasinghe et al. 2019; Guo et al. 2020; Hilário et al. 2021a; Xiao et al. 2023). For instance, 19 *Diaporthe* species were reported to be associated with pear shoot canker in China, and Koch’s postulates confirmed that all were pathogenic. Among them, *D. chongqingensis*, *D. fusicola*, and *D. eres* were highly aggressive (Guo et al. 2020). Xiao et al. (2023) reported 36 *Diaporthe* species associated with citrus disease; the pathogenicity test revealed that *D. citri* produced the longest lesions, followed by *D. tectonae*, *D. hubeiensis*, *D. sexualispora*, and *D. citriasiana*. Eight *Diaporthe* species were reported in association with grapevine dieback, and *D. gulyae* was the most aggressive taxon, followed by *D. eres* and *D. unshiuensis* (Manawasinghe et al. 2019). In Portugal, Hilário et al. (2021a) found that *D. eres* and *D. amygdali* were the most virulent species associated with blueberry twig blight and dieback. These results indicate that the pathogenicity of the same species can vary depending on the host. For instance, *D. eres* was highly aggressive on walnut, pear, and blueberry, but considerably less so on citrus.

## ﻿Conclusion

In conclusion, this study presents the *Diaporthe* species associated with branch diseases of walnuts in China. A total of seven species, including one novel species, were identified, all of which were confirmed as causal agents of walnut branch blight and dieback. This study also revealed the diversity and geographical distribution of *Diaporthe* spp. associated with walnut. The findings provide valuable insight into the ecology and pathogenicity of *Diaporthe* spp. involved in walnut blight and dieback.

## Supplementary Material

XML Treatment for Diaporthe amygdali

XML Treatment for Diaporthe citrichinensis

XML Treatment for Diaporthe eres

XML Treatment for Diaporthe psoraleae-pinnatae

XML Treatment for Diaporthe rostrata

XML Treatment for Diaporthe sackstonii

XML Treatment for Diaporthe yunnana

## ﻿Additional information

### Use of AI

No use of AI was reported.

### Author ORCIDs

Lili Zhao https://orcid.org/0000-0003-1451-3301

Lin Zhang https://orcid.org/0009-0002-6325-1440

Ying Zhang https://orcid.org/0000-0001-8817-6032

### Data availability

All of the data that support the findings of this study are available in the main text or Supplementary Information.

## Supplementary materials

Supplementary material 1 GenBank accession numbers of isolates included in this study Data type xlsx https://binary.pensoft.net/file/1413891This dataset is made available under the Open Database License (http://opendatacommons.org/licenses/odbl/1.0/). The Open Database License (ODbL) is a license agreement intended to allow users to freely share, modify, and use this Dataset while maintaining this same freedom for others, provided that the original source and author(s) are credited.Lili Zhao, Lin Zhang, Yi Ding, Ming Li, Ying Zhang

Supplementary material 2 Morphological characteristics of *D. amygdali* Data type jpg https://binary.pensoft.net/file/1413892This dataset is made available under the Open Database License (http://opendatacommons.org/licenses/odbl/1.0/). The Open Database License (ODbL) is a license agreement intended to allow users to freely share, modify, and use this Dataset while maintaining this same freedom for others, provided that the original source and author(s) are credited.Lili Zhao, Lin Zhang, Yi Ding, Ming Li, Ying Zhang

Supplementary material 3 Morphological characteristics of *D. citrichinensis* Data type jpg https://binary.pensoft.net/file/1413893This dataset is made available under the Open Database License (http://opendatacommons.org/licenses/odbl/1.0/). The Open Database License (ODbL) is a license agreement intended to allow users to freely share, modify, and use this Dataset while maintaining this same freedom for others, provided that the original source and author(s) are credited.Lili Zhao, Lin Zhang, Yi Ding, Ming Li, Ying Zhang

Supplementary material 4 Morphological characteristics of *D. eres* Data type jpg https://binary.pensoft.net/file/1413894This dataset is made available under the Open Database License (http://opendatacommons.org/licenses/odbl/1.0/). The Open Database License (ODbL) is a license agreement intended to allow users to freely share, modify, and use this Dataset while maintaining this same freedom for others, provided that the original source and author(s) are credited.Lili Zhao, Lin Zhang, Yi Ding, Ming Li, Ying Zhang

Supplementary material 5 Morphological characteristics of *D. psoraleae-pinnatae* Data type jpg https://binary.pensoft.net/file/1413895This dataset is made available under the Open Database License (http://opendatacommons.org/licenses/odbl/1.0/). The Open Database License (ODbL) is a license agreement intended to allow users to freely share, modify, and use this Dataset while maintaining this same freedom for others, provided that the original source and author(s) are credited.Lili Zhao, Lin Zhang, Yi Ding, Ming Li, Ying Zhang

Supplementary material 6 Morphological characteristics of *D. rostrata* Data type jpg https://binary.pensoft.net/file/1413896This dataset is made available under the Open Database License (http://opendatacommons.org/licenses/odbl/1.0/). The Open Database License (ODbL) is a license agreement intended to allow users to freely share, modify, and use this Dataset while maintaining this same freedom for others, provided that the original source and author(s) are credited.Lili Zhao, Lin Zhang, Yi Ding, Ming Li, Ying Zhang

Supplementary material 7 Morphological characteristics of *D. sackstonii* Data type jpg https://binary.pensoft.net/file/1413897This dataset is made available under the Open Database License (http://opendatacommons.org/licenses/odbl/1.0/). The Open Database License (ODbL) is a license agreement intended to allow users to freely share, modify, and use this Dataset while maintaining this same freedom for others, provided that the original source and author(s) are credited.Lili Zhao, Lin Zhang, Yi Ding, Ming Li, Ying Zhang

## References

[B1] Calabon MS Jones EBG Pang KL Abdel-Wahab MA Jin J Devadatha B Sadaba RB Apurillo CC Hyde KD (2023) Updates on the classification and numbers of marine fungi. Botanica Marina 66(4): 213–238. 10.1515/bot-2023-0032

[B2] Cao JY Gao WK Yao M Xie SP Yin XM Xu C Wu HY Zhang M Guo YS (2023) *Diaporthe actinidiicola*: A novel species causing branch canker or dieback of fruit trees in Henan Province, China. Plant Pathology 72(7): 1236–1246. 10.1111/ppa.13744

[B3] Carbone I Kohn LM (1999) A method for designing primer sets for speciation studies in filamentous ascomycetes. Mycologia 91: 553–556. 10.2307/3761358

[B4] Chen SF Morgan DP Hasey JK Anderson K Michailides TJ (2014) Phylogeny, morphology, distribution, and pathogenicity of Botryosphaeriaceae and Diaporthaceae from English walnut in California. Plant Disease 98(5): 636–652. 10.1094/PDIS-07-13-0706-RE 30708543

[B5] Crous PW Wingfield MJ Guarro J Cheewangkoon R van der Bank M Swart WJ Stchigel AM Cano-Lira JF Roux J Madrid H Damm U Wood AR Shuttleworth LA Hodges CS Munster M de Jesús Yáñez-Morales M Zúñiga-Estrada L Cruywagen EM De Hoog GS Silvera C Najafzadeh J Davison EM Davison PJN Barrett MD Barrett RL Ma namgoda DS Minnis AM Kleczewski NM Flory SL Castlebury LA Clay K Hyde KD Maússe-Sitoe SND Chen Shuaifei Lechat C Hairaud M Lesage-Meessen L Pawłowska J Wilk M Śliwińska-Wyrzychowska A Mętrak M Wrzosek M Pavlic-Zupanc D Maleme HM Slippers B Mac Cormack WP Archuby DI Grünwald NJ Tellería MT Dueñas M Martín MP Marincowitz S de Beer ZW Perez CA Gené J Marin-Felix Y Groenewald JZ (2013) Fungal planet description sheets: 154–213. Persoonia 31(1): 188–296. 10.3767/003158513X675925 24761043 PMC3904050

[B6] Dela Cruz FM Bermeo-Capunong MRA Bagacay JFE Canto CM Calabon MS (2025) Taxonomy, phylogeny, and preliminary screening of fungal isolates for cadmium tolerance from Iloilo Ferry Terminal, Iloilo, Philippines. Studies in Fungi 10: e001. 10.48130/sif-0025-0001

[B7] Delacroix G (1905) Sur une maladie des Amendiers en Provence. Bulletin de la Société Mycologique de France. 21: 180–185.

[B8] Dissanayake AJ Zhu JT Chen YY Maharachchikumbura SSN Hyde KD Liu JK (2024) A re-evaluation of *Diaporthe*: Refining the boundaries of species and species complexes. Fungal Diversity 126(1): 127–406. 10.1007/s13225-024-00538-7

[B9] Eichmeier A Pecenka J Spetik M Necas T Ondrasek I Armengol J León M Berlanas C Gramaje D (2020) Fungal trunk pathogens associated with *Juglans regia* in the Czech Republic. Plant Disease 104(3): 761–771. 10.1094/PDIS-06-19-1308-RE 31944904

[B10] Fan XL Hyde KD Udayanga D Wu XY Tian CM (2015) *Diaporthe rostrata*, a novel ascomycete from *Juglans mandshurica* associated with walnut dieback. Mycological Progress 14(10): 82. 10.1007/s11557-015-1104-5

[B11] Fan XL Yang Q Bezerra JDP Alvarez LV Tian CM (2018) *Diaporthe* from walnut tree (*Juglans regia*) in China, with insight of the *Diaporthe eres* complex. Mycological Progress 17(7): 841–853. 10.1007/s11557-018-1395-4

[B12] Fu M Crous PW Bai Q Zhang PF Xiang J Guo YS Zhao FF Yang MM Hong N Xu WX Wang GP (2019) *Colletotrichum* species associated with anthracnose of *Pyrus* spp. in China. Persoonia 42: 1–35. 10.3767/persoonia.2019.42.01 31551612 PMC6712541

[B13] Glass NL Donaldson GC (1995) Development of primer sets designed for use with the PCR to amplify conserved genes from filamentous ascomycetes. Applied and Environmental Microbiology 61: 1323–1330. 10.1128/aem.61.4.1323-1330.1995 7747954 PMC167388

[B14] Gomes RR Glienke C Videira SIR Lombard L Groenewald JZ Grous PW (2013) *Diaporthe*: A genus of endophytic, saprobic and plant pathogenic fungi. Persoonia 31: 1–41. 10.3767/003158513X666844 24761033 PMC3904044

[B15] Guo YS Crous PW Bai Q Fu M Yang MM Wang XH Du YM Hong N Xu WX Wang GP (2020) High diversity of *Diaporthe* species associated with pear shoot canker in China. Persoonia 45: 132–162. 10.3767/persoonia.2020.45.05 34456374 PMC8375346

[B16] Hilário S Gonçalves MFM (2023) Mechanisms underlying the pathogenic and endophytic lifestyles in *Diaporthe*: An omics-based approach. Horticultural 9(4): 423. 10.3390/horticulturae9040423

[B17] Hilário S Santos L Alves A (2021a) Diversity and pathogenicity of *Diaporthe* species revealed from a survey of blueberry orchards in Portugal. Agriculture-Basel 11(12): 1271. 10.3390/agriculture11121271

[B18] Hilário S Gonçalves MFM Alves A (2021b) Using genealogical concordance and coalescent-based species delimitation to assess species boundaries in the *Diaporthe eres* complex. Journal of Fungi 7: 507. 10.3390/jof7070507 PMC8307253 34202282

[B19] Hillis DM Bull JJ (1993) An empirical test of bootstrapping as a method for assessing confidence in phylogenetic analysis. Systematic Biology 42(2): 182–192. 10.1093/sysbio/42.2.182

[B20] Huang F Hou X Dewdney MM Fu Y Chen GQ Hyde KD Li HY (2013) *Diaporthe* species occurring on *Citrus* in China. Fungal Diversity 61(1): 237–250. 10.1007/s13225-013-0245-6

[B21] Jia AL Lin L Li YX Fan XL (2024) Diversity and pathogenicity of six *Diaporthe* species from *Juglans regia* in China. Journal of Fungi 10(8): 583. 10.3390/jof10080583 39194908 PMC11355219

[B22] Katoh K Rozewicki J Yamada KD (2019) MAFFT online service: Multiple sequence alignment, interactive sequence choice and visualization. Briefings in Bioinformatics 20(4): 1160–1166. 10.1093/bib/bbx108 28968734 PMC6781576

[B23] Li JT Li Y Li JR Jiang N (2025) Species of *Diaporthe* (Diaporthaceae, Diaporthales) associated with *Alnus nepalensis* leaf spot and branch canker diseases in Xizang, China. MycoKeys 116: 185–204. 10.3897/mycokeys.116.142750 40313691 PMC12044343

[B24] López-Moral A Lovera M Raya MD Cortes-Cosano N Arquero O Trapero A Agustí-Brisach C (2020) Etiology of branch dieback and shoot blight of English walnut caused by Botryosphaeriaceae and *Diaporthe* species in southern Spain. Plant Disease 104(2): 533–550. 10.1094/PDIS-03-19-0545-RE 31746696

[B25] López-Moral A Lovera M Antón-Domínguez BI Michailides TJ Arquero O Trapero A Agustí-Brisach C (2023) Effects of cultivar susceptibility, fruit maturity, and natural wounds on the infection of English walnut (*Juglans regia* L.) fruits by Botryosphaeriaceae and *Diaporthe* fungi. Journal of Plant Pathology 105(4): 1391–1401.

[B26] Luna IJ Cadiz F Aravena R Larach A Besoain X Ezcurra E Rolshausen PE (2020) First Report of *Diaporthe cynaroides* and *D. australafricana* associated with walnut branch canker in Chile. Plant Disease 104(10): 2732–2733. 10.1094/PDIS-01-20-0205-PDN

[B27] Luna IJ Doll D Ashworth VETM Trouillas FP Rolshausen PE (2023) Comparative profiling of wood canker pathogens from spore traps and symptomatic plant samples within California almond and walnut orchards. Plant Disease 106(8): 2182–2190. 10.1094/PDIS-05-21-1057-RE 35077222

[B28] Manawasinghe IS Dissanayake AJ Li XH Liu M Wanasinghe DN Xu JP Zhao WS Zhang W Zhou YY Hyde KD Brooks S Yan JY (2019) High genetic diversity and species complexity of *Diaporthe* associated with grapevine dieback in China. Frontiers in Microbiology 10: 1936. 10.3389/fmicb.2019.01936 PMC6732904 31543868

[B29] Meng L Yu C Wang C Li G (2018) First report of *Diaporthe amygdali* causing walnut twig canker in Shandong province of China. Plant Disease 102: 1859. 10.1094/PDIS-01-18-0192-PDN

[B30] Nitschke T (1870) Pyrenomycetes Germanici 2. Eduard Trewendt, Breslau, 245 pp.

[B31] Norphanphoun C Gentekaki E Hongsanan S Jayawardena R Senanayake IC Manawasinghe IS Abeywickrama PD Bhunjun CS Hyde KD (2022) *Diaporthe*: Formalizing the species-group concept. Mycosphere 13(1): 752–819. 10.5943/mycosphere/13/1/9

[B32] Page RD (1996) TREEVIEW: An application to display phylogenetic trees on personal computers. Computer Applications in the Biosciences 12: 357–358. 10.1093/bioinformatics/12.4.357 8902363

[B33] Pereira DA Phillips AJL (2024) *Diaporthe* species on palms – integrative taxonomic approach for species boundaries delimitation in the genus *Diaporthe*, with the description of *D. pygmaeae* sp. nov. Studies in Mycology 109: 487–594. 10.3114/sim.2024.109.08 39717652 PMC11663421

[B34] Posada D Buckley TR (2004) Model selection and model averaging in phylogenetics: Advantages of Akaike information criterion and Bayesian approaches over likelihood ratio tests. Systematic Biology 53: 793–808. 10.1080/10635150490522304 15545256

[B35] Rayner RW (1970) A mycological colour chart. Commonwealth Mycological Institute, Kew, UK.

[B36] Ronquist F Teslenko M van der Mark P Ayres DL Darling A Höhna S Larget B Liu L Suchard MA Huelsenbeck JP (2012) MrBayes 3.2: Efficient bayesian phylogenetic inference and model choice across a large model space. Systematic Biology 61: 539–542. 10.1093/sysbio/sys029 22357727 PMC3329765

[B37] Rossman AY Adams GC Cannon PF Castlebury LA Crous PW Gryzenhout M Jaklitsch WM Mejia LC Stoykov D Udayanga D Voglmayr H Walker DM (2015) Recommendations of generic names in Diaporthales competing for protection or use. IMA Fungus 6(1): 145–154. 10.5598/imafungus.2015.06.01.09 26203420 PMC4500080

[B38] Shen DY Wu ST Zheng YM Han YX Ni ZL Li SL Tang FB Mo RH Liu YH (2021) Characterization of iron walnut in different regions of China based on phytochemical composition. Journal of Food Science and Technology-Mysore. 58(4): 1358–1367. 10.1007/s13197-020-04647-4 PMC7925734 33746264

[B39] Stamatakis A (2014) RAxML version 8: A tool for phylogenetic analysis and post analysis of large phylogenies. Bioinformatics 30: 1312–1313. 10.1093/bioinformatics/btu033 24451623 PMC3998144

[B40] Sun W Huang S Xia J Zhang X Li Z (2021) Morphological and molecular identification of *Diaporthe* species in south-western China, with description of eight new species. MycoKeys 77: 65–95. 10.3897/mycokeys.77.59852 33519269 PMC7819953

[B41] Swofford DL (2002) PAUP*: phylogenetic analysis using parsimony (*and other methods). Version 4.0b10. Sinauer Associates, Sunderland, Massachusetts.

[B42] Tamura K Stecher G Peterson D Filipski A Kumar S (2013) MEGA6: Molecular evolutionary genetics analysis version 6.0. Molecular Biology and Evolution 30: 2725–2729. 10.1093/molbev/mst197 24132122 PMC3840312

[B43] Thompson SM Tan YP Shivas RG Neate SM Morin L Bissett A Aitken EAB (2015) Green and brown bridges between weeds and crops reveal novel *Diaporthe* species in Australia. Persoonia 35: 39–49. 10.3767/003158515X687506 26823627 PMC4713110

[B44] Ting T Yang CD Cai FF Osei R (2023) Molecular Identification and Characterization of *Fusarium* associated with walnut branch blight disease in China. Pathogens 12(7): 970. 10.3390/pathogens12070970 37513816 PMC10384706

[B45] Udayanga D Liu X Crous PW McKenzie EH Chukeatirote E Chukeatirote E Hyde KD (2012) A multi-locus phylogenetic evaluation of Diaporthe (Phomopsis). Fungal Diversity 56(1): 157–171. 10.1007/s13225-012-0190-9

[B46] Udayanga D Castlebury LA Rossman AY Chukeatirote E Hyde KD (2014) Insights into the genus *Diaporthe*: Phylogenetic species delimitation in the *D. eres* species complex. Fungal Diversity 67(1): 203–229. 10.1007/s13225-014-0297-2

[B47] Uecker FA (1988) A world list of *Phomopsis* names with notes on nomenclature, morphology and biology. Mycological Memoirs 13: 1–231.

[B48] Wang SY McKenzie EHC Phillips AJL Li Y Wang Y (2022) Taxonomy and multigene phylogeny of Diaporthales in Guizhou province, China. Journal of Fungi 8(12): 1301. 10.3390/jof8121301 36547633 PMC9785342

[B49] White TJ Bruns T Lee S Taylor JL (1990) Amplification and direct sequencing of fungal ribosomal RNA genes for phylogenetics. PCR Protocols: a guide to methods and applications 18: 315–322. 10.1016/B978-0-12-372180-8.50042-1

[B50] Xiao XE Liu YD Zheng F Xiong T Zeng YT Wang W Zeng XL Wu Q Xu JP Crous PW Jiao C Li HY (2023) High species diversity in *Diaporthe* associated with citrus diseases in China. Persoonia 51: 229–256. 10.3767/persoonia.2023.51.06 38665984 PMC11041894

[B51] Yang Q Fan XL Guarnaccia V Tian CM (2018) High diversity of *Diaporthe* species associated with dieback diseases in China, with twelve new species described. MycoKeys 39: 97–149. 10.3897/mycokeys.39.26914 PMC6160862 30271260

[B52] Zhang R Tao L (2025) Analysis of the production and trade status of walnut in China. Zhongguo Guoshu 1: 147–152. 10.16626/j.cnki.issn1000-8047.2025.01.022

[B53] Zhao LL Sun W Zhang L Yin YQ Xie YQ Zhang Y (2024) Heart rot disease of walnut caused by *Nothophoma juglandis* sp. nov. and its endophytic biocontrol agent. Plant Disease 108(3): 746–756. 10.1094/PDIS-11-22-2660-RE 37787687

