# Supplementary figures and images for: ﻿Diaporthe species (Sordariomycetes, Diaporthales) causing walnut blight and dieback in China
Source: MycoKeys. 2025 Sep 12;122:197–221. doi: 10.3897/mycokeys.122.158807 (PMC12449697; doi:10.3897/mycokeys.122.158807)

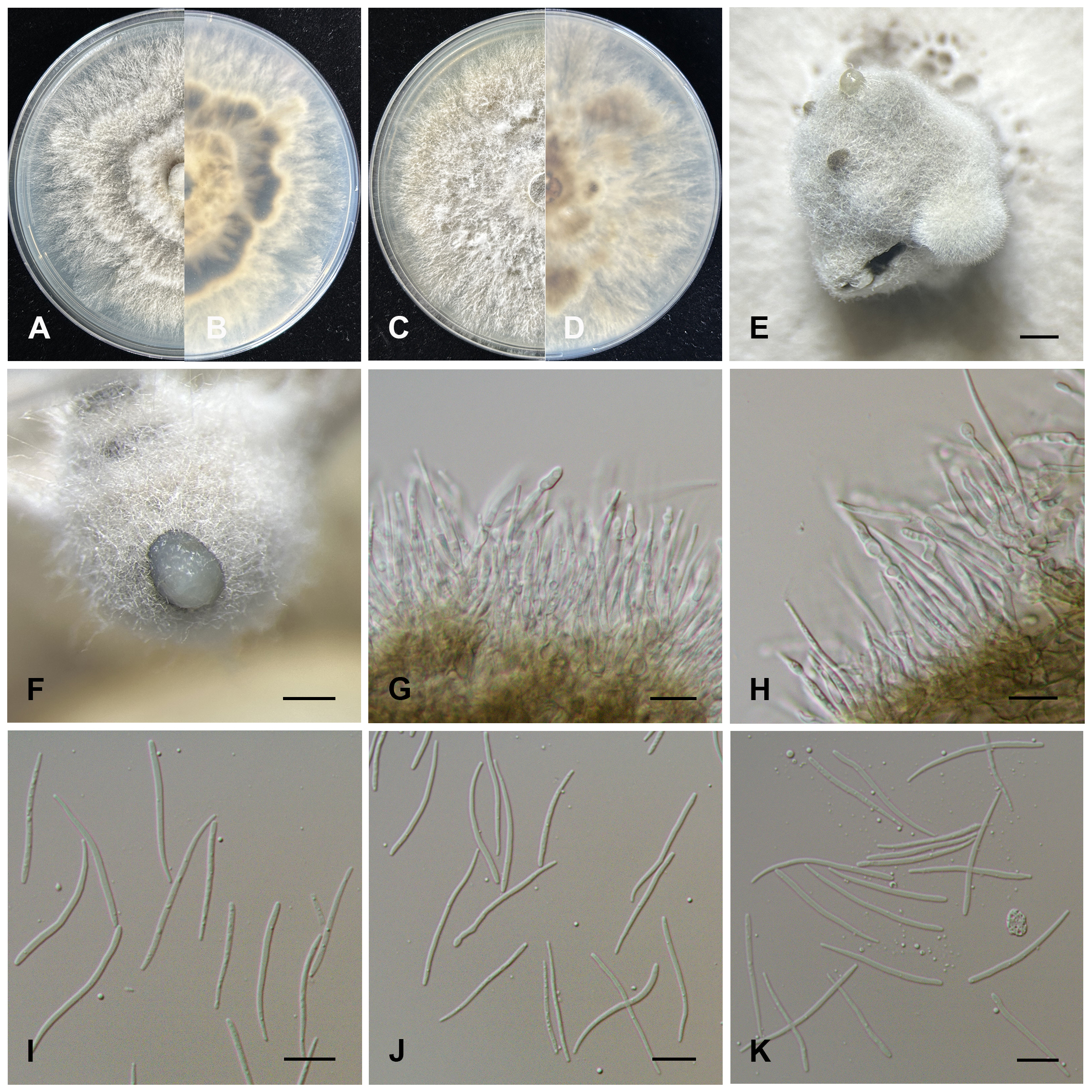

Supplement: Supplementary material 2 — Morphological characteristics of D. amygdali [file mycokeys-122-197-s002.jpg]

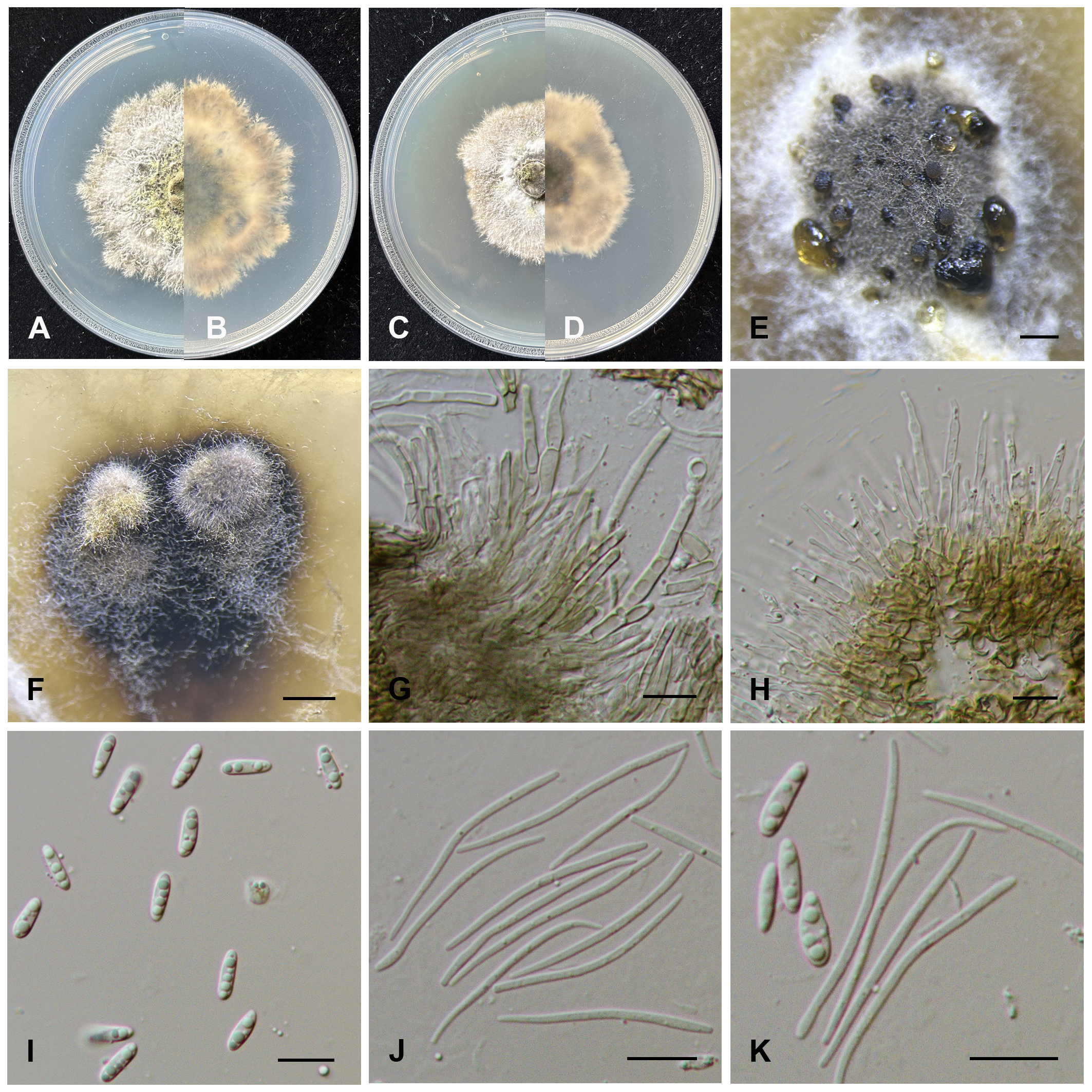

Supplement: Supplementary material 3 — Morphological characteristics of D. citrichinensis [file mycokeys-122-197-s003.jpg]

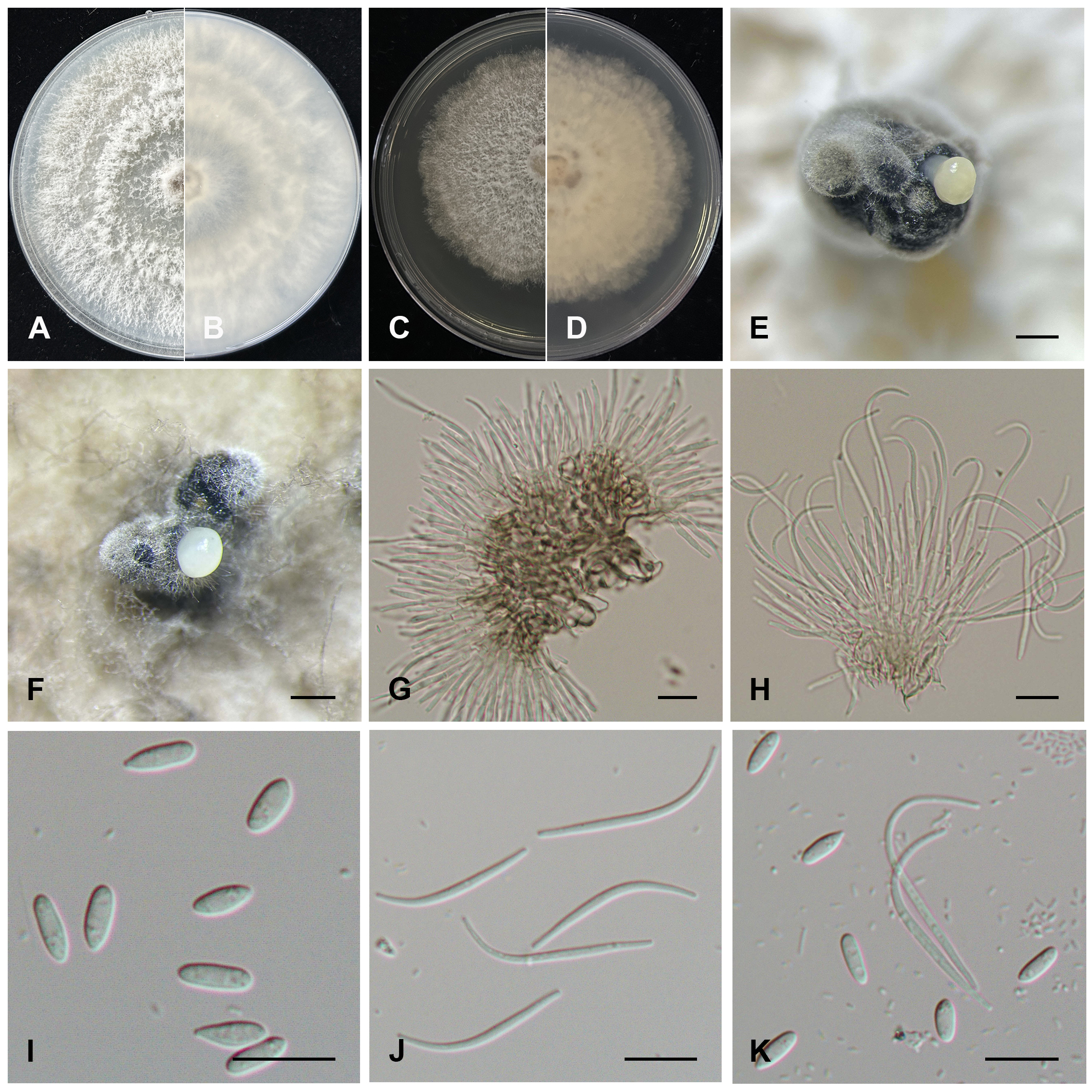

Supplement: Supplementary material 4 — Morphological characteristics of D. eres [file mycokeys-122-197-s004.jpg]

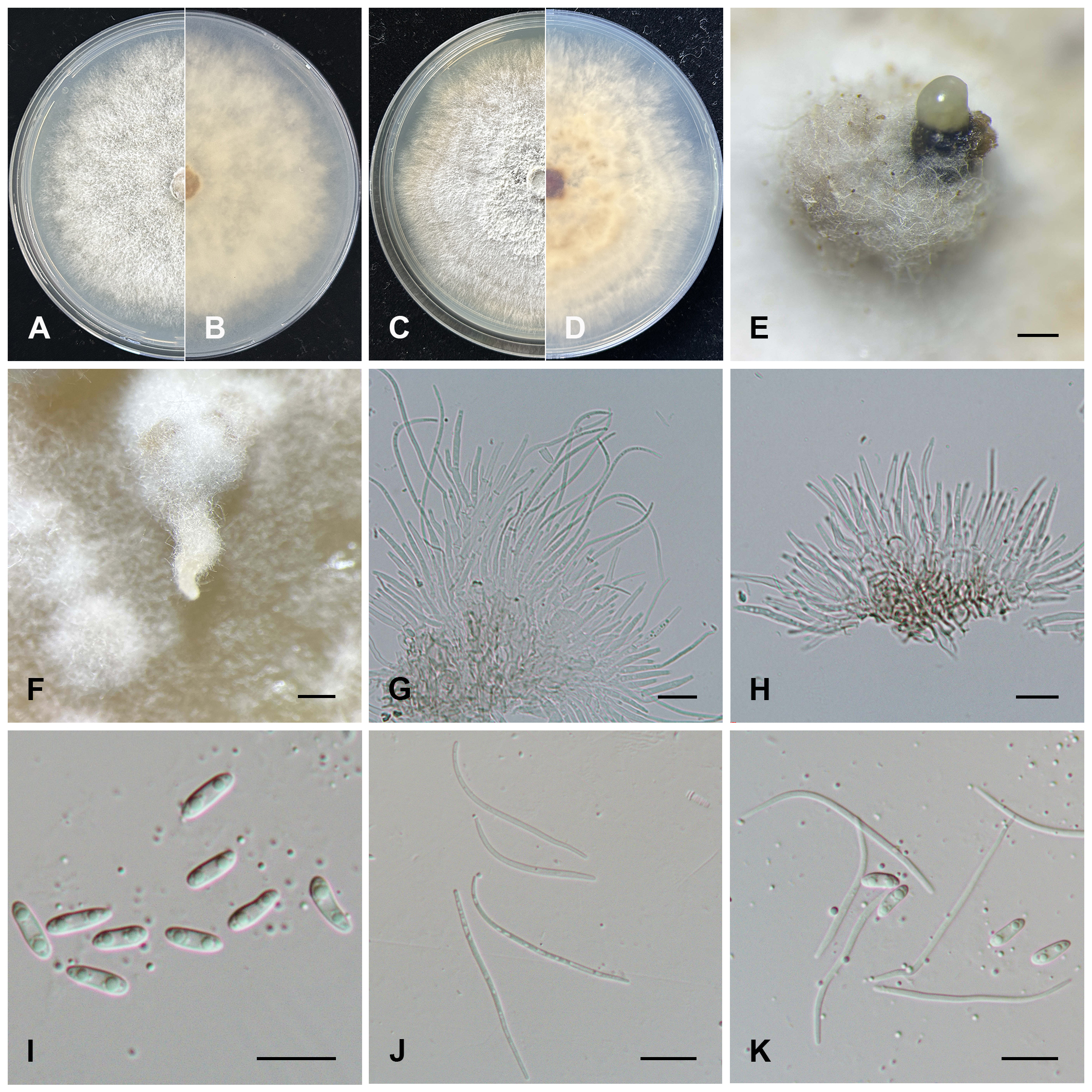

Supplement: Supplementary material 5 — Morphological characteristics of D. psoraleae-pinnatae [file mycokeys-122-197-s005.jpg]

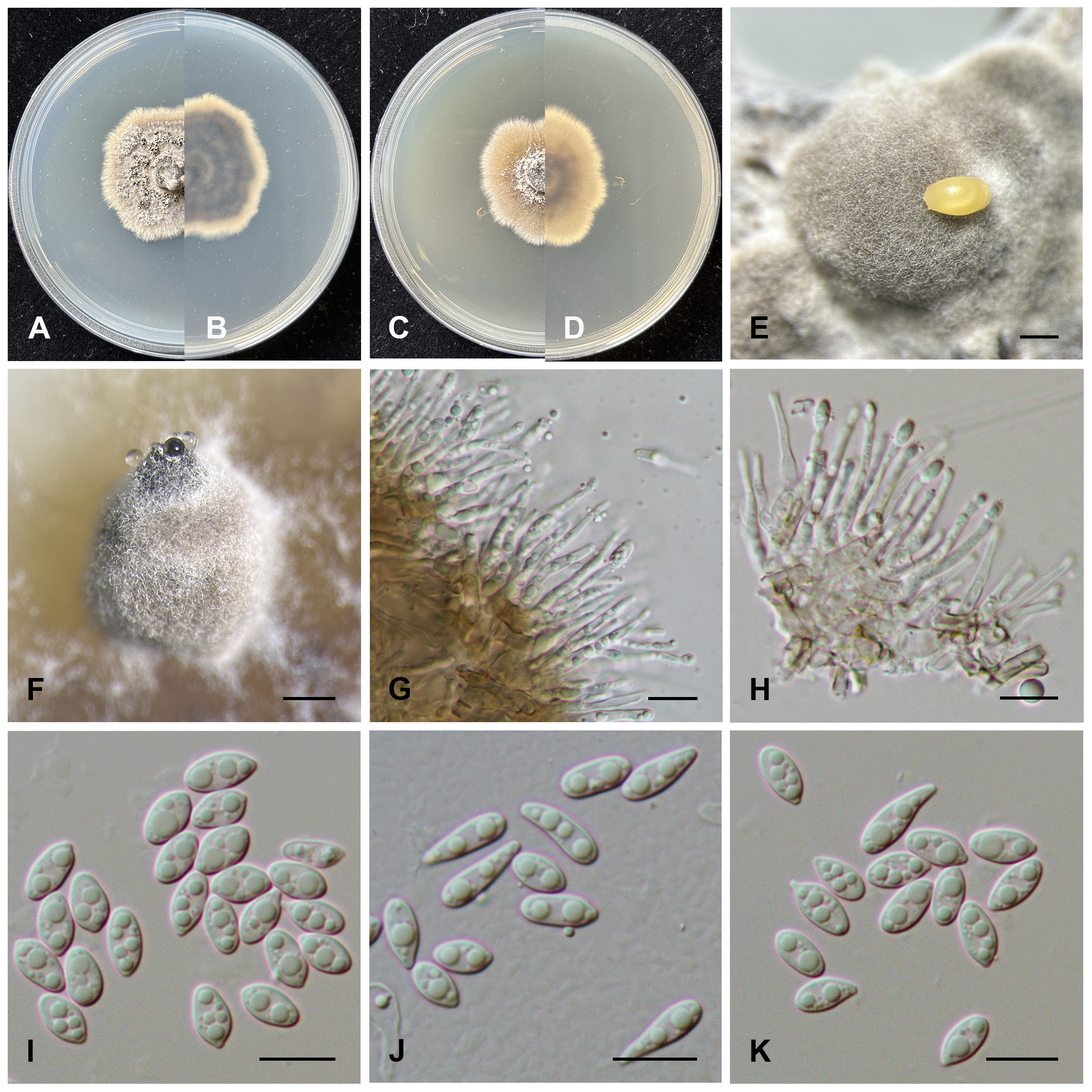

Supplement: Supplementary material 6 — Morphological characteristics of D. rostrata [file mycokeys-122-197-s006.jpg]

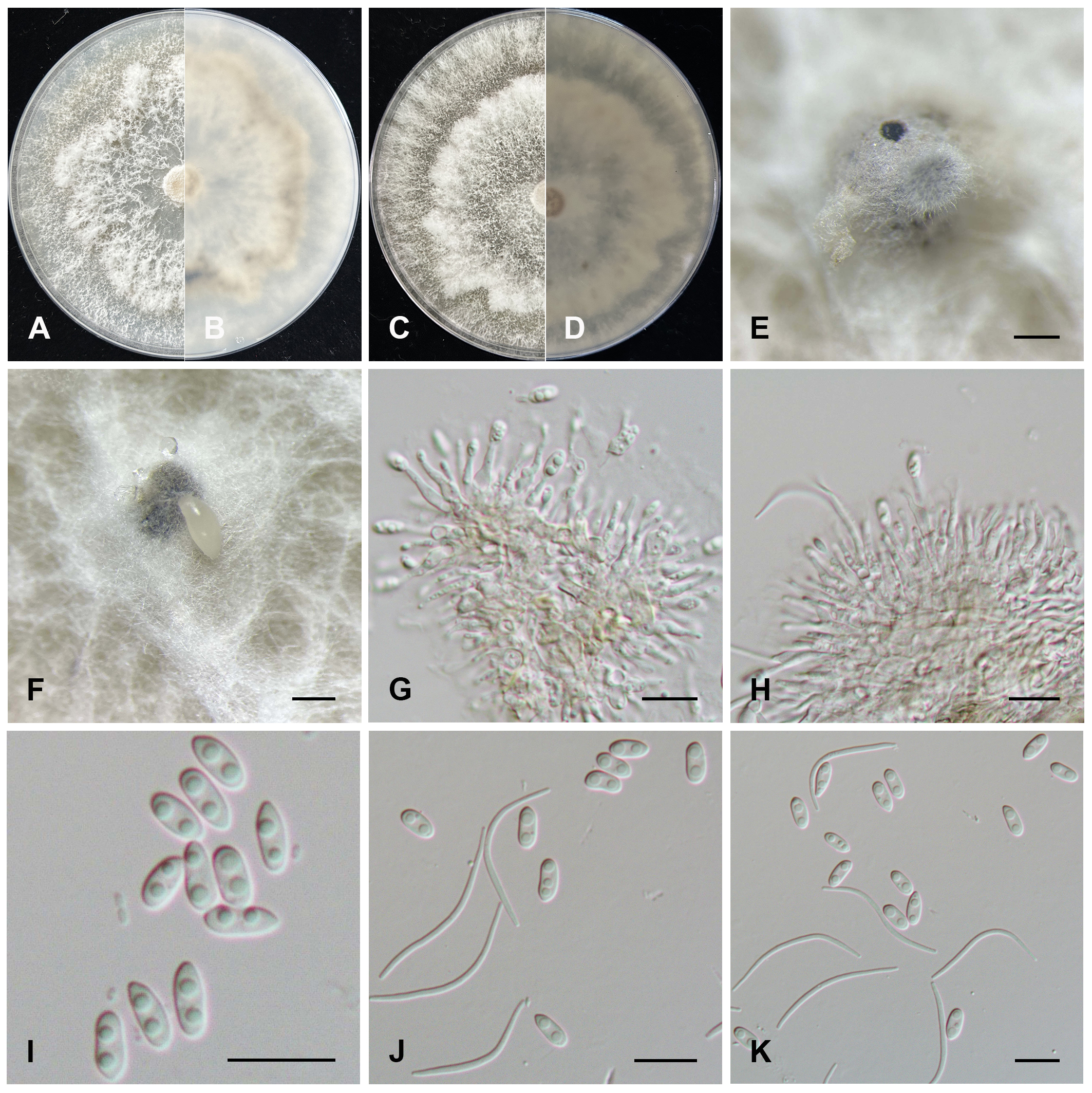

Supplement: Supplementary material 7 — Morphological characteristics of D. sackstonii [file mycokeys-122-197-s007.jpg]
